# Limited Predictive Value of Concentric Knee and Trunk Isokinetic Peak Torque for Maximal Force and Stretch-Shortening Cycle Performance in Highly Trained Youth Optimist Sailors

**DOI:** 10.3390/jfmk11030374

**Published:** 2026-09-20

**Authors:** Radomyos Matjiur, Piyathida Thongchai, Sadanan Kernluea, Phornpot Chainok, Rodrigo Zacca

**Affiliations:** 1 Faculty of Sport Science, Burapha University, Chonburi 20131, Thailand; radomyos.ma@go.buu.ac.th (R.M.); piyathida.th@go.buu.ac.th (P.T.); soxphiax16@gmail.com (S.K.); 2 Research Center in Physical Activity, Health and Leisure (CIAFEL), Faculty of Sports, University of Porto (FADEUP), 4200-450 Porto, Portugal; rzacca@fade.up.pt; 3 Laboratory for Integrative and Translational Research in Population Health (ITR), 4050-600 Porto, Portugal; 4 Laboratory of Sport Physiology, Faculty of Sports, University of Porto, 4200-450 Porto, Portugal; 5 Nucleus of Research in Human Motricity Sciences, Universidad Adventista de Chile, Chillan 3780000, Chile

**Keywords:** sailing, strength qualities, core body strength, strength and power continuum, neuromuscular performance, youth athletes

## Abstract

**Background:** Concentric knee and trunk strength may contribute differently to maximal force and stretch-shortening cycle performance in youth sailors. This study evaluated the predictive value of concentric knee and trunk isokinetic strength for these outcomes in highly trained youth Optimist sailors. **Methods:** Forty highly trained Optimist sailors (20 boys and 20 girls; age, 13.03 ± 1.40 years) completed knee and trunk isokinetic testing at 60°·s^−1^ and 180°·s^−1^, isometric mid-thigh pull, squat jump, and countermovement jump tests. Velocity-specific multiple linear regression models included a composite of dominant and nondominant knee extensor and flexor strength, trunk extensor strength, trunk flexor strength, and sex. **Results:** The models explained 60.7% and 49.4% of the variance in isometric mid-thigh pull peak force at 60°·s^−1^ and 180°·s^−1^, respectively. The knee strength composite was independently associated with peak force at both velocities (β = 0.51, *p* = 0.003; β = 0.56, *p* = 0.006). The models explained 1.3–3.5% of dynamic strength index variance and 17.8% and 4.5% of eccentric utilization ratio and elastic index variance at 60°·s^−1^ and 180°·s^−1^, respectively. Trunk flexor strength was associated with eccentric utilization ratio and elastic index at 60°·s^−1^ (β = 0.52, *p* = 0.029), although these outcomes are mathematically dependent. Sex was not associated with any outcome. **Conclusions:** Concentric isokinetic strength showed greater predictive value for maximal force than for stretch-shortening cycle performance. Combined knee extensor and flexor strength was consistently associated with maximal force, whereas limited variance was explained in stretch-shortening cycle outcomes.

## 1. Introduction

Optimist sailing race performance is primarily determined by environmental conditions, tactical execution and boat–handling proficiency, physical fitness is a key determinant of the ability to sustain effective boat control during prolonged hiking, repeated maneuvering and continuous postural stabilization [1,2,3]. Hiking, the predominant physical demand of competitive sailing, requires sustained force generation by the knee extensors, trunk flexor and extensor muscles and hip musculature to counteract heeling forces and maintain an optimal sailing position [4,5,6].

Consistent with these demands, hiking-specific training has been shown to enhance muscular function and cardiorespiratory capacity in highly trained sailors, emphasizing the importance of developing sport-specific strength capacities [7]. As youth sailors undergo concurrent biological maturation and athletic development, identifying the strength characteristics that underpin sailing performance is essential for informing evidence-based training prescription and optimizing long-term athlete development [8,9,10,11,12]. Therefore, both factors should be considered when investigating the relationships between muscle strength and neuromuscular performance in youth athletes.

Muscular strength constitutes a fundamental component of athletic performance because it provides the mechanical foundation for force production, power expression, movement efficiency and resilience to training and competition demands [13]. In youth athletes, systematic resistance training produces robust improvements in maximal strength, power, sprinting, jumping and sport-specific performance, although these adaptations are influenced by biological maturation, which substantially affects neuromuscular development during adolescence [14,15,16,17,18,19]. Consequently, precise assessment of distinct strength qualities is essential for understanding performance capabilities and guiding individualized training interventions. Isokinetic dynamometry is considered the gold standard for assessing dynamic muscle strength, providing valid and reliable measures of joint torque under controlled angular velocities [20]. This assessment is particularly relevant to sailing, where optimal performance depends on the capacity of the knee and trunk musculature to generate high joint torques during maneuvers and maintain postural stability during prolonged hiking [5,6].

Likewise, force–platform assessments, including the isometric mid-thigh pull (IMTP), squat jump (SJ) and countermovement jump (CMJ), are widely used to quantify maximal isometric force production, explosive strength and stretch-shortening cycle (SSC) function through reliable measures of neuromuscular performance [14,21,22,23,24]. The dynamic strength index (DSI) reflects the relationship between dynamic and maximal isometric force expression, whereas the eccentric utilization ratio (EUR) and elastic index (EI) provide further insight into an athlete’s ability to translate maximal strength into dynamic force production and effectively utilize eccentric loading during stretch-shortening cycle (SSC) activities [25,26]. However, the physiological interpretation of some derived indices, particularly EUR, remains inconsistent across athletic populations [27,28,29].

Despite the established importance of knee and trunk strength for sustaining hiking performance in sailing [4,5,6,7], it remains unclear whether isolated isokinetic strength reflects whole-body force production and stretch-shortening cycle (SSC)-related neuromuscular characteristics in highly trained youth Optimist sailors. Clarifying these relationships may improve the interpretation of complementary neuromuscular assessments used for athlete profiling and monitoring. Therefore, this cross-sectional study evaluated the predictive value of concentric knee and trunk isokinetic peak torque for maximal force and stretch-shortening cycle performance, represented by IMTP peak force, DSI, EUR and EI, after adjustment for sex in highly trained youth Optimist sailors.

We hypothesized that concentric knee and trunk isokinetic peak torque would demonstrate greater predictive value for maximal force, represented by IMTP peak force, than for stretch-shortening cycle performance, represented by DSI, EUR and EI, after adjustment for sex. This hypothesis was based on the premise that maximal force production is primarily constrained by concentric force-generating capacity, whereas stretch-shortening cycle performance depends on the integrated contribution of neuromuscular, mechanical and coordinative factors that are not fully captured by isolated concentric isokinetic strength.

## 2. Materials and Methods

### 2.1. Participants

Sample size was estimated a priori using G*Power (version 3.1.9.2) to ensure adequate statistical power. Assuming a large effect size (Cohen’s f^2^ = 0.95), an α level of 0.05, and statistical power (1 − β) of 0.95, the minimum required sample size was 30 participants. The effect-size assumption was informed by previous studies in youth sailing reporting substantial associations between performance and physical, anthropometric and sailing-related characteristics [1,2]. Accordingly, forty highly trained youth Optimist sailors were recruited to participate in this cross-sectional study, comprising 20 boys (13.31 ± 0.85 years) and 20 girls (12.75 ± 1.76 years), with a total mean age of 13.03 ± 1.40 years. Based on the participant classification framework proposed by McKay et al. [27], 16 sailors were classified as Tier 4 (international level), having represented the national team and competed at the Optimist World Championship, whereas the remaining 24 sailors were classified as Tier 3 (national level). Eligible participants had ≥2 years of competitive Optimist sailing experience and were actively competing at the national or international level. Participants were excluded if they had a recent musculoskeletal injury, previous surgery affecting physical performance, or any neurological or medical condition that could compromise maximal testing performance.

### 2.2. Procedures

**Figure 1:**
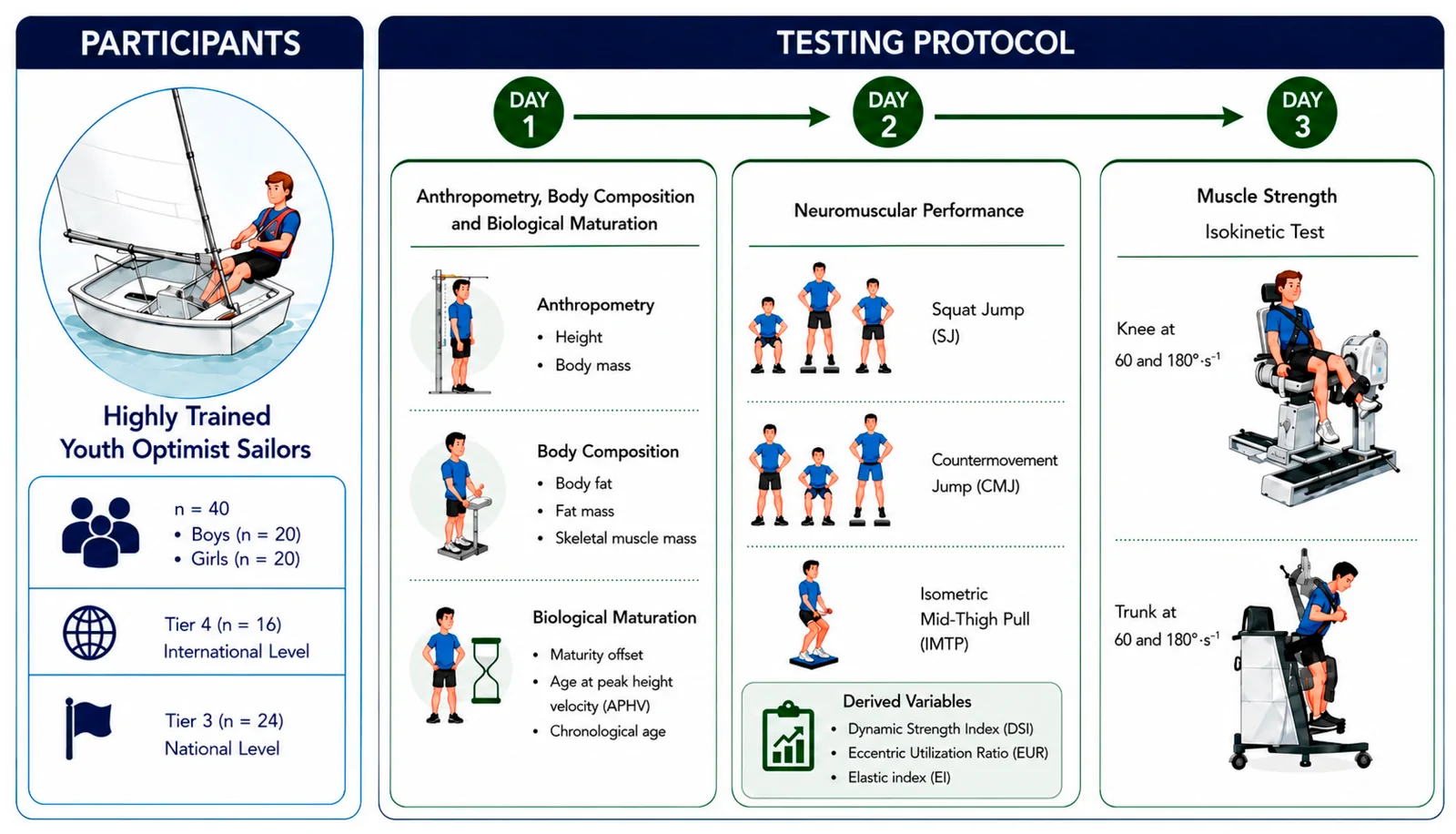
Experimental design and three-day testing protocol for highly trained youth Optimist sailors. Image created by ChatGPT (GPT-5.6 Luna).

All testing was completed over three consecutive days in a temperature-controlled laboratory (25–27 °C, 50–60% relative humidity) (Figure 1). Each participant was assessed at the same time of day to minimize circadian variation. Participants were instructed to refrain from strenuous exercise for 24 h before each testing session and to maintain their habitual dietary and hydration practices throughout the study. On the first day, anthropometric characteristics, body composition and biological maturation were assessed. On the second day, lower-limb neuromuscular performance was evaluated using force–platform assessments, including the SJ, CMJ and IMTP. The DSI, EUR and EI were subsequently calculated from the force–platform data. On the third day, concentric isokinetic strength of the knee flexors, knee extensors, trunk flexors and trunk extensors was assessed at angular velocities of 60°·s^−1^ and 180°·s^−1^ using an isokinetic dynamometer. All testing procedures were supervised by the same investigators, and standardized verbal encouragement was provided during every maximal-effort trial.

### 2.3. Anthropometric Characteristics and Biological Maturation

Standing height was measured using an ultrasonic stadiometer, whereas body mass, body mass index (BMI), body fat percentage, fat mass and skeletal muscle mass were determined by multifrequency bioelectrical impedance analysis (X–Contact 357S, Jawon Medical, Gyeongsan, Republic of Korea). Biological maturation was estimated using the sex-specific maturity offset equations proposed by Moore et al. [30], from which maturity offset (years from peak height velocity) and predicted age at peak height velocity (APHV) were calculated. Chronological age was calculated from the participant’s date of birth to the testing date.

### 2.4. Lower-Limb Neuromuscular Performance

Lower-limb neuromuscular performance was assessed using dual force platforms (K–Deltas, Kinvent Physio, Montpellier, France; sampling frequency: 1000 Hz). Following a standardized warm-up, participants completed three maximal trials of the SJ, CMJ and IMTP in a randomized order, with 2 min of passive recovery between trials. For all force-time variables, the highest value obtained across the three maximal trials was retained for statistical analysis. For the SJ, participants assumed a self-selected squat position, maintained the position for approximately 2–3 s to minimize stretch-shortening cycle contribution and performed a maximal vertical jump with their hands positioned on the hips [22]. The CMJ was performed from an upright standing position using a rapid countermovement immediately followed by a maximal vertical jump while maintaining the hands on the hips [23]. The IMTP was performed using a fixed bar with participants positioned in the second-pull posture of the clean and instructed to pull maximally against the immovable bar for 5 s [21].

Relative total peak force (N·kg^−1^) obtained during the CMJ and IMTP was used to calculate the dynamic strength index (DSI) as the ratio of CMJ to IMTP peak force [24,25]. The eccentric utilization ratio (EUR) was calculated as the ratio of CMJ to SJ jump height to quantify stretch-shortening cycle (SSC) utilization during vertical jumping [28,29]. The elastic index (EI) was calculated as the percentage increase in CMJ jump height relative to SJ jump height, representing the additional performance attributable to SSC utilization [31]. All force–time variables were processed using the manufacturer’s software and verified prior to statistical analysis.

### 2.5. Isokinetic Strength Assessment

Concentric isokinetic strength of the knee and trunk musculature was assessed using an Isoforce dynamometer (TUR GmbH, Berlin, Germany). Peak torque of the dominant and nondominant knee extensors and flexors and the trunk extensors and flexors was measured at angular velocities of 60°·s^−1^ and 180°·s^−1^. Before data collection, participants completed a standardized warm-up followed by familiarization trials at each testing velocity. Five maximal concentric repetitions were subsequently completed for each muscle group at both angular velocities, with 60 s of passive recovery between testing conditions. For each muscle group and angular velocity, the highest peak torque was retained and normalized to body mass (N·m·kg^−1^) for statistical analysis. Torque signals were sampled at 1000 Hz, processed using the manufacturer’s software, and exported for subsequent analysis [21].

### 2.6. Statistical Analysis

Statistical analyses were performed using IBM SPSS Statistics (Version 28; IBM Corp., Armonk, NY, USA). Data were screened for outliers, and missing values and normality was assessed using the Shapiro–Wilk test. Continuous variables are presented as mean ± standard deviation (SD). The within-session reliability of isokinetic and force-platform variables was assessed using the intraclass correlation coefficient (ICC), coefficient of variation (CV), and standard error of measurement (SEM). ICCs were calculated using a two-way random-effects model with absolute agreement and single measures [ICC (2,1)]. Between-sex differences were evaluated using independent-samples t-tests, with Hedges’ g calculated to quantify effect sizes. Associations between relative knee and trunk isokinetic peak torque and neuromuscular performance variables (IMTP peak force, DSI, EUR and EI) were examined using Pearson’s product-moment correlation coefficients. To control the family-wise error rate, Holm’s sequential Bonferroni correction was applied to the correlation analyses.

Separate velocity-specific multiple linear regression analyses were conducted at 60°·s^−1^ and 180°·s^−1^. For each testing velocity, a knee strength composite was calculated as the mean of the z-standardized relative peak torque values of the dominant knee extensors, nondominant knee extensors, dominant knee flexors, and nondominant knee flexors. The knee strength composite, trunk extensor strength, trunk flexor strength, and sex were entered simultaneously as predictors of IMTP peak force, DSI, EUR EI. Separate models were constructed for each testing velocity using the corresponding relative isokinetic peak torque measures. Model results are reported as standardized regression coefficients (β), 95% confidence intervals, *p*-values, R^2^, adjusted R^2^, standard error of the estimate and variance inflation factors. Model assumptions were evaluated using residual diagnostics, multicollinearity was assessed using variance inflation factors, and influential observations were identified using Cook’s distance. Statistical significance was accepted at *p* < 0.05.

## 3. Results

Descriptive characteristics of the participants are presented in Table 1. The study included 40 highly trained youth Optimist sailors (20 boys and 20 girls) with a mean age of 13.03 ± 1.40 years. Boys were, on average, older (13.31 ± 0.85 vs. 12.75 ± 1.76 years) and taller (156.86 ± 6.52 vs. 153.90 ± 8.56 cm) than girls, whereas girls exhibited greater body mass (45.92 ± 7.97 vs. 41.12 ± 5.88 kg), body mass index (17.79 ± 3.44 vs. 16.93 ± 2.63 kg·m^−2^), body fat percentage (16.15 ± 5.61 vs. 9.89 ± 7.93%), fat mass (7.43 ± 3.19 vs. 3.99 ± 3.55 kg) and maturity offset (0.54 ± 1.26 vs. 0.20 ± 0.85 years). Boys had greater skeletal muscle mass than girls (21.30 ± 3.70 vs. 19.77 ± 3.97 kg). The estimated age at peak height velocity was comparable between sexes (boys: 12.71 ± 0.83 years; girls: 12.86 ± 0.78 years).

The within-session reliability of the force-platform and isokinetic variables is presented in Table 2. Overall, ICC values ranged from 0.59 to 0.94, with coefficients of variation ranging from 0.76% to 10.91%. Among the force-platform variables, SJ peak force (ICC = 0.88, 95% CI: 0.78–0.94) and CMJ peak force (ICC = 0.89, 95% CI: 0.84–0.93) demonstrated the highest reliability, whereas SJ height, CMJ height and IMTP peak force demonstrated ICC values ranging from 0.77 to 0.80. The neuromuscular performance indices exhibited lower reliability than the direct force measures, with ICC values of 0.71 for DSI and 0.59 for both EUR and EI. For the isokinetic variables, ICC values ranged from 0.82 to 0.90 at 60°·s^−1^ for the trunk measures and from 0.86 to 0.88 for the knee measures. At 180°·s^−1^, ICC values ranged from 0.71 to 0.94, with the trunk extensor demonstrating the highest reliability (ICC = 0.94, 95% CI: 0.91–0.96; CV = 0.76%) and the dominant knee flexor the lowest (ICC = 0.71, 95% CI: 0.60–0.81; CV = 8.47%). Coefficients of variation for the isokinetic variables ranged from 0.76% to 10.91%, while SEM values varied according to the measurement scale of each variable.

Sex differences in neuromuscular performance and isokinetic peak torque are presented in Table 3. Boys demonstrated significantly greater lower-limb explosive performance than girls, achieving higher SJ height (20.77 ± 2.53 vs. 18.30 ± 2.50 cm, *p* = 0.003, g = 0.96), CMJ height (22.55 ± 2.95 vs. 19.87 ± 2.81 cm, *p* = 0.005, g = 0.91) and CMJ peak force (25.55 ± 3.99 vs. 22.70 ± 3.37 N·kg^−1^, *p* = 0.019, g = 0.76). In contrast, SJ peak force, IMTP peak force, DSI, EUR and EI did not differ significantly between sexes (*p* = 0.102–0.941), with trivial-to-moderate effect sizes (g = −0.12 to 0.52). A consistent pattern of greater isokinetic peak torque was observed in boys. At 60°·s^−1^, boys produced significantly greater nondominant knee extensor (*p* = 0.019, g = 0.76), dominant knee flexor (*p* = 0.032, g = 0.69) and nondominant knee flexor peak torque (*p* = 0.002, g = 1.04), whereas the difference in dominant knee extensor peak torque approached statistical significance (*p* = 0.059, g = 0.60). No significant sex differences were observed for trunk extensor or trunk flexor peak torque at this angular velocity (*p* = 0.105 and 0.472, respectively).

At 180°·s^−1^, boys demonstrated significantly greater peak torque across all knee muscle groups, including the dominant knee extensor (*p* = 0.001, *g* = 1.08), nondominant knee extensor (*p* = 0.009, *g* = 0.86), dominant knee flexor (*p* = 0.005, *g* = 0.92) and nondominant knee flexor (*p* < 0.001, *g* = 1.16). Similarly, trunk extensor (*p* = 0.002, *g* = 1.02) and trunk flexor peak torque (*p* = 0.017, *g* = 0.78) were significantly greater in boys than girls. Overall, effect sizes ranged from trivial to large (g = −0.12 to 1.16). The largest between-sex differences were observed for nondominant knee flexor peak torque at 180°·s^−1^ (g = 1.16), dominant knee extensor peak torque at 180°·s^−1^ (g = 1.08), nondominant knee flexor peak torque at 60°·s^−1^ (g = 1.04) and trunk extensor peak torque at 180°·s^−1^ (g = 1.02).

**Figure 2:**
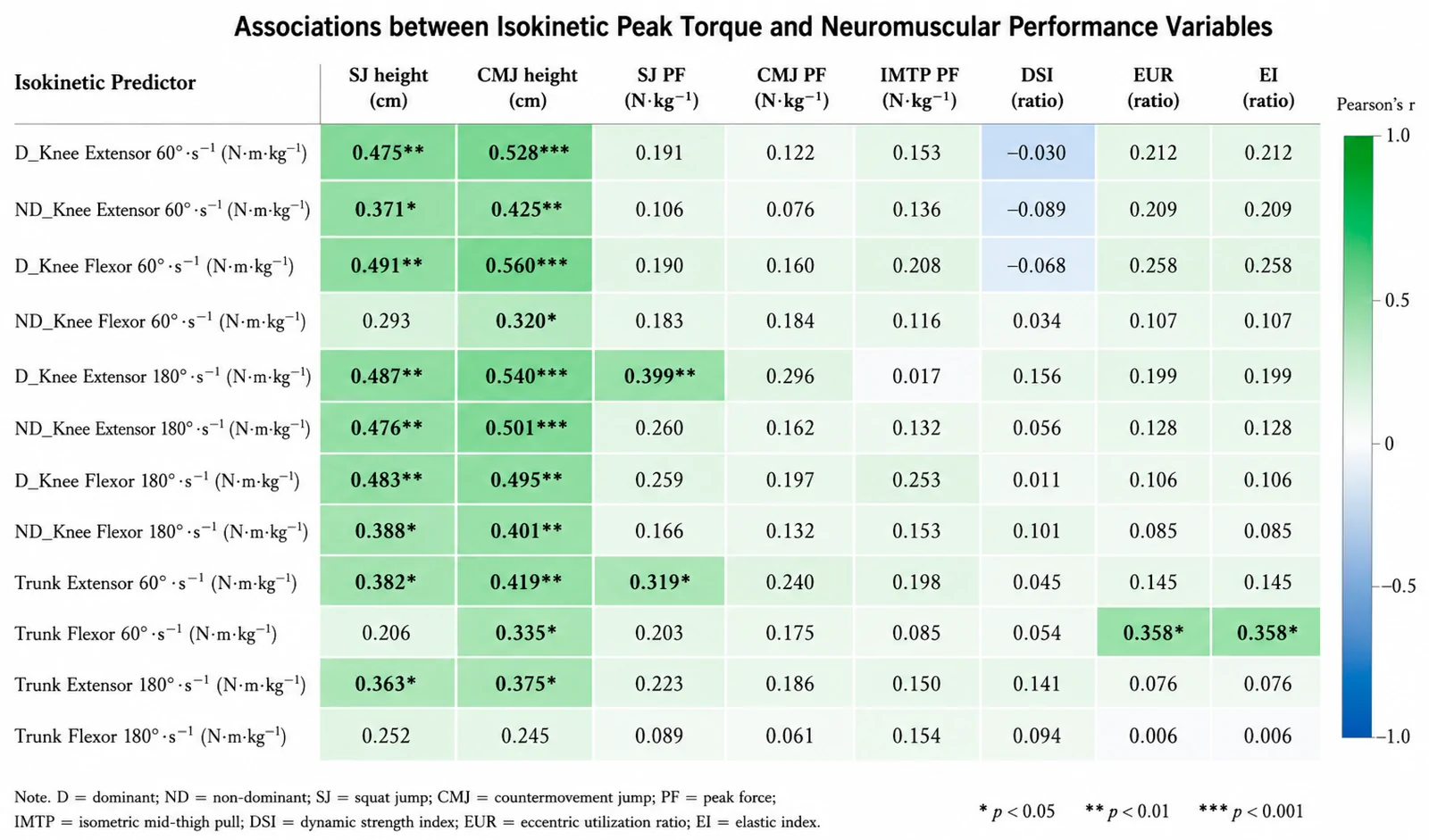
Correlation heatmap showing associations between knee and trunk isokinetic peak torque and lower-limb neuromuscular performance variables. Statistical significance is based on Holm’s sequential Bonferroni-adjusted *p*-values: * *p* < 0.05, ** *p* < 0.01, and *** *p* < 0.001.

Figure 2 presents the associations between concentric knee and trunk isokinetic peak torque and neuromuscular performance variables. SJ height was positively associated with dominant knee extensor peak torque at both 60°·s^−1^ (r = 0.475, *p* < 0.01) and 180°·s^−1^ (r = 0.487, *p* < 0.01), dominant knee flexor peak torque at 60°·s^−1^ (r = 0.491, *p* < 0.01) and 180°·s^−1^ (r = 0.483, *p* < 0.01), nondominant knee extensor peak torque at 60°·s^−1^ (r = 0.371, *p* < 0.05) and 180°·s^−1^ (r = 0.476, *p* < 0.01), nondominant knee flexor peak torque at 180°·s^−1^ (r = 0.388, *p* < 0.05), trunk extensor peak torque at 60°·s^−1^ (r = 0.382, *p* < 0.05) and 180°·s^−1^ (r = 0.363, *p* < 0.05) and trunk flexor peak torque at 60°·s^−1^ (r = 0.335, *p* < 0.05). A similar pattern was observed for CMJ height, which demonstrated significant associations with most knee isokinetic measures (r = 0.320–0.560, *p* < 0.05–0.001) and trunk extensor peak torque at both 60°·s^−1^ (r = 0.419, *p* < 0.01) and 180°·s^−1^ (r = 0.375, *p* < 0.05), whereas trunk flexor peak torque at 60°·s^−1^ was also significantly associated with CMJ height (r = 0.335, *p* < 0.05).

Among the force-based variables, SJ peak force was significantly associated only with dominant knee extensor peak torque at 180°·s^−1^ (r = 0.399, *p* < 0.01) and trunk extensor peak torque at 60°·s^−1^ (r = 0.319, *p* < 0.05). No significant associations were observed between any isokinetic peak torque measure and CMJ peak force or IMTP peak force, with correlation coefficients ranging from 0.017 to 0.253 for IMTP peak force. For the stretch-shortening cycle-derived variables, DSI was not significantly associated with any isokinetic peak torque measure. Significant associations were observed only between trunk flexor peak torque at 60°·s^−1^ and both EUR (r = 0.358, *p* < 0.05) and EI (r = 0.358, *p* < 0.05). No other significant associations were identified for EUR or EI.

The velocity-specific multiple regression analyses are presented in Table 4. The knee strength composite was positively and independently associated with isometric mid-thigh pull peak force at both 60°·s^−1^ (β = 0.51, 95% CI: 0.19–0.84, *p* = 0.003) and 180°·s^−1^ (β = 0.56, 95% CI: 0.17–0.96, *p* = 0.006). No significant independent associations were observed for dynamic strength index at either velocity. At 60°·s^−1^, trunk flexor strength was positively associated with eccentric utilization ratio and elastic index (β = 0.52, 95% CI: 0.06–0.98, *p* = 0.029), whereas no significant associations were observed for these outcomes at 180°·s^−1^. Sex was not independently associated with any outcome.

Model fit and diagnostic statistics are presented in Table 5. The isometric mid-thigh pull peak force models showed the greatest explanatory capacity, accounting for 60.7% of the variance at 60°·s^−1^ (adjusted R^2^ = 0.562) and 49.4% at 180°·s^−1^ (adjusted R^2^ = 0.436). The dynamic strength index models explained little variance (R^2^ = 0.013–0.035), while the eccentric utilization ratio and elastic index models explained 17.8% of the variance at 60°·s^−1^ and 4.5% at 180°·s^−1^. Maximum VIF values were 3.36 at 60°·s^−1^ and 4.18 at 180°·s^−1^, indicating that severe multicollinearity was not evident in the revised models. Maximum Cook’s distance ranged from 0.09 to 0.29 across models, with no observation exceeding 1.0.

## 4. Discussion

The present study evaluated the predictive value of concentric knee and trunk isokinetic peak torque for maximal force and stretch-shortening cycle performance in highly trained youth Optimist sailors. The main finding was that concentric isokinetic strength showed greater predictive value for maximal force than for stretch-shortening cycle performance. The knee strength composite was independently associated with isometric mid-thigh pull peak force at both 60°·s^−1^ and 180°·s^−1^ after adjustment for sex, whereas the models explained substantially less variance in dynamic strength index, eccentric utilization ratio, and elastic index. Trunk flexor strength at 60°·s^−1^ was associated with eccentric utilization ratio and elastic index, which represent mathematically dependent expressions of the same countermovement jump–squat jump relationship.

The correlation analyses were consistent with these findings, showing only small associations between selected knee and trunk muscle groups and IMTP peak force, whereas associations with DSI, EUR and EI were sparse and generally weak. Boys exhibited greater jump performance and higher isokinetic peak torque than girls, particularly at 180°·s^−1^; however, sex did not independently contribute to any regression model. Collectively, these findings support the hypothesis that concentric knee and trunk isokinetic peak torque has greater predictive value for maximal force than for stretch-shortening cycle performance, while indicating that concentric isokinetic strength alone provides limited insight into neuromuscular performance in highly trained youth Optimist sailors.

The knee strength composite was independently associated with isometric mid-thigh pull peak force at both testing velocities after adjustment for sex. The models explained 60.7% of the variance at 60°·s^−1^ and 49.4% at 180°·s^−1^, indicating substantially greater explanatory capacity for maximal force than for the stretch-shortening cycle-derived outcomes. Isokinetic dynamometry assesses regional joint torque under controlled conditions [20], whereas the isometric mid-thigh pull requires coordinated whole-body force transmission through the lower limbs and trunk [21]. The moderate variance inflation observed at 180°·s^−1^ warrants some caution when interpreting individual coefficients, although severe multicollinearity was not evident in the revised models.

From a sport-specific perspective, these findings are compatible with the physical demands of Optimist sailing, where prolonged hiking requires sustained force production by the knee extensors together with continuous trunk stabilization to counter external heeling moments and maintain sailing posture [4,5,6,7]. Previous studies have also identified lower-limb strength and hiking capacity as important determinants of sailing performance [1,4]. Therefore, knee and trunk strength should be considered an important physical quality for youth sailors, although it represents only one component of maximal force production [13,14].

Considering sex differences and maturation, boys demonstrated greater jump performance and higher isokinetic peak torque than girls, particularly at 180°·s^−1^, whereas no sex differences were observed for IMTP peak force, DSI, EUR, or EI. Furthermore, sex did not independently predict any neuromuscular outcome after adjustment for isokinetic strength. These findings agree with evidence that neuromuscular performance during adolescence is influenced primarily by biological maturation and muscle development rather than chronological age or sex alone [9,10,11,12,19]. The descriptive characteristics of the present cohort support this interpretation, as boys had greater skeletal muscle mass, whereas girls demonstrated a greater maturity offset despite a similar estimated age at peak height velocity. Consequently, biological maturation should be considered when evaluating physical performance and prescribing strength training in youth sailors [12,16,17,20].

In contrast to isometric mid-thigh pull peak force, concentric knee and trunk isokinetic peak torque showed limited predictive value for stretch-shortening cycle-derived outcomes. No strength variable independently predicted dynamic strength index, whereas trunk flexor strength at 60°·s^−1^ was associated with eccentric utilization ratio and elastic index. Stretch-shortening cycle performance is influenced by multiple neuromuscular and mechanical factors, including rapid force production, intermuscular coordination, tendon behaviour, and the interaction between eccentric and concentric actions [25,26,30,31]. However, eccentric utilization ratio and elastic index represent the same underlying countermovement jump–squat jump relationship because elastic index is a deterministic transformation of eccentric utilization ratio. Accordingly, these findings should be interpreted as a single pattern of association rather than as independent evidence from two neuromuscular outcomes. This interpretation is also consistent with previous research questioning the use of eccentric utilization ratio as an isolated indicator of stretch-shortening cycle function [26,28]. The limited variance explained by these models indicates that isolated concentric strength provides relatively little information regarding the countermovement jump–squat jump performance relationship.

The limited predictive value observed in the present study is also consistent with the movement characteristics of Optimist sailing. Sailing performance depends on prolonged quasi-isometric hiking interspersed with rapid whole-body movements during maneuvers, requiring coordinated force production rather than isolated concentric muscle strength [3,5,6]. Consequently, force-platform and isokinetic assessments should be regarded as complementary measures, with the former evaluating whole-body neuromuscular performance and the latter quantifying regional muscle strength [20,23]. From a practical perspective, the findings suggest that resistance training for youth Optimist sailors should continue to develop knee and trunk strength because these muscle groups contribute to maximal force production and hiking performance [4,5,6,7,13]. However, improvements in stretch-shortening cycle performance are unlikely to be achieved through concentric strength development alone. Training programmes should therefore combine maximal strength training with ballistic, plyometric, and sailing-specific exercises to develop rapid force production and stretch-shortening cycle function [14,15,16,17,24].

There are several limitations that should be considered when interpreting the present findings. The cross-sectional design prevents causal inferences and the relatively small sample size may have limited statistical power to detect small associations, especially for outcomes based on the stretch-shortening cycle. Also, the a priori sample size calculation was based on a relatively large effect size which may have limited the sensitivity of the study to detect smaller associations. Thus, the non-significant results for DSI, EUR and EI should be interpreted with caution. Biological maturation and body-size characteristics were not included in the regression models and may have explained the variation in neuromuscular performance observed in individual adolescents. In addition, the measures in the laboratory were not directly related to water performance in sailing. Future prospective studies with larger samples should consider maturation and body-size normalization and include sailing-specific performance measures to clarify the practical relevance of concentric knee and trunk strength in youth sailors.

## 5. Conclusions

The present study demonstrated that concentric knee and trunk isokinetic peak torque has limited predictive value for maximal force and stretch-shortening cycle performance in highly trained youth Optimist sailors. Although knee extensor and trunk extensor peak torque independently predicted IMTP peak force after adjustment for sex, the explained variance was modest. In contrast, concentric isokinetic peak torque did not independently predict DSI, EUR, or EI, indicating that isolated concentric strength provides limited insight into stretch-shortening cycle performance. These findings suggest that isokinetic dynamometry, IMTP and jump-based assessments evaluate complementary neuromuscular qualities and should be used together when profiling youth sailors. Accordingly, training programmes should combine knee and trunk strength development with exercises targeting explosive force production and stretch-shortening cycle function.

## Figures and Tables

**Table 1:** Descriptive characteristics of the participants by sex.

Variables	Boys (n = 20)	Girls (n = 20)	Total (n = 40)
Age (years)	13.31 ± 0.85	12.75 ± 1.76	13.03 ± 1.40
Standing height (cm)	156.86 ± 6.52	153.90 ± 8.56	155.38 ± 7.66
Body mass (kg)	41.12 ± 5.88	45.92 ± 7.97	43.52 ± 7.33
Body mass index (kg·m^−2^)	16.93 ± 2.63	17.79 ± 3.44	17.36 ± 3.06
Body fat (%)	9.89 ± 7.93	16.15 ± 5.61	13.02 ± 7.48
Fat mass (kg)	3.99 ± 3.55	7.43 ± 3.19	5.71 ± 3.76
Skeletal muscle mass (kg)	21.30 ± 3.70	19.77 ± 3.97	20.54 ± 3.87
Maturity offset (years)	0.20 ± 0.85	0.54 ± 1.26	0.37 ± 1.07
Age at peak height velocity (years)	12.71 ± 0.83	12.86 ± 0.78	12.79 ± 0.80

**Table 2:** Within-session reliability of force–platform and isokinetic variables.

Variables	ICC (95% CI)	CV (%)	SEM
SJ height (cm)	0.78 (0.69–0.86)	4.16	0.81
CMJ height (cm)	0.80 (0.71–0.87)	3.38	0.73
SJ_PF (N·kg^−1^)	0.88 (0.78–0.94)	2.16	0.61
CMJ_PF (N·kg^−1^)	0.89 (0.84–0.93)	5.21	1.18
IMTP_PF (N·kg^−1^)	0.77 (0.67–0.85)	9.02	2.71
DSI (ratio)	0.71 (0.60–0.81)	6.45	0.05
EUR (ratio)	0.59 (0.45–0.72)	4.08	0.05
EI (ratio)	0.59 (0.45–0.72)	5.02	4.62
D_Knee Extensor 60°·s^−1^ (N·m·kg^−1^)	0.87 (0.80–0.91)	5.30	0.19
ND_Knee Extensor 60°·s^−1^ (N·m·kg^−1^)	0.87 (0.80–0.91)	8.80	0.15
D_Knee Flexor 60°·s^−1^ (N·m·kg^−1^)	0.88 (0.82–0.92)	10.91	0.07
ND_Knee Flexor 60°·s^−1^ (N·m·kg^−1^)	0.86 (0.81–0.92)	2.91	0.07
D_Knee Extensor 180°·s^−1^ (N·m·kg^−1^)	0.78 (0.69–0.86)	9.75	0.23
ND_Knee Extensor 180°·s^−1^ (N·m·kg^−1^)	0.76 (0.68–0.85)	9.40	0.21
D_Knee Flexor 180°·s^−1^ (N·m·kg^−1^)	0.71 (0.60–0.81)	8.47	0.13
ND_Knee Flexor 180°·s^−1^ (N·m·kg^−1^)	0.79 (0.72–0.85)	7.14	0.16
Trunk Extensor 60°·s^−1^ (N·m·kg^−1^)	0.82 (0.74–0.88)	8.00	7.13
Trunk Flexor 60°·s^−1^ (N·m·kg^−1^)	0.90 (0.86–0.94)	5.00	4.07
Trunk Extensor 180°·s^−1^ (N·m·kg^−1^)	0.94 (0.91–0.96)	0.76	0.48
Trunk Flexor 180°·s^−1^ (N·m·kg^−1^)	0.89 (0.83–0.93)	7.49	4.17

Note: SJ = squat jump; CMJ = counter movement jump; IMTP = isometric mid-thigh pull; PF = peak force; DSI = dynamic strength index; EUR = eccentric utilization ratio; EI = elastic index; D = dominant; ND = nondominant.

**Table 3:** Sex differences in neuromuscular and isokinetic variables.

Variables	Boys (n = 20)	Girls (n = 20)	*p*	Hedges’ *g*
SJ height (cm)	20.77 ± 2.53	18.30 ± 2.50	0.003	0.96
CMJ height (cm)	22.55 ± 2.95	19.87 ± 2.81	0.005	0.91
SJ PF (N·kg^−1^)	23.47 ± 2.86	22.12 ± 2.81	0.145	0.47
CMJ PF (N·kg^−1^)	25.55 ± 3.99	22.70 ± 3.37	0.019	0.76
IMTP PF (N·kg^−1^)	36.50 ± 8.34	32.23 ± 7.78	0.102	0.52
DSI (ratio)	0.72 ± 0.16	0.74 ± 0.17	0.774	−0.12
EUR (ratio)	1.09 ± 0.05	1.09 ± 0.07	0.941	0.00
EI (ratio)	8.56 ± 5.31	8.71 ± 7.17	0.941	−0.02
D_Knee Extensor 60°·s^−1^ (N·m·kg^−1^)	2.21 ± 0.52	1.91 ± 0.45	0.059	0.60
ND_Knee Extensor 60°·s^−1^ (N·m·kg^−1^)	2.00 ± 0.42	1.69 ± 0.38	0.019	0.76
D_Knee Flexor 60°·s^−1^ (N·m·kg^−1^)	1.52 ± 0.36	1.26 ± 0.38	0.032	0.69
ND_Knee Flexor 60°·s^−1^ (N·m·kg^−1^)	1.28 ± 0.28	1.00 ± 0.25	0.002	1.04
D_Knee Extensor 180°·s^−1^ (N·m·kg^−1^)	1.67 ± 0.42	1.24 ± 0.37	0.001	1.08
ND_Knee Extensor 180°·s^−1^ (N·m·kg^−1^)	1.46 ± 0.39	1.15 ± 0.32	0.009	0.86
D_Knee Flexor 180°·s^−1^ (N·m·kg^−1^)	1.19 ± 0.36	0.89 ± 0.27	0.005	0.92
ND_Knee Flexor 180°·s^−1^ (N·m·kg^−1^)	1.00 ± 0.30	0.72 ± 0.16	<0.001	1.16
Trunk Extensor 60°·s^−1^ (N·m·kg^−1^)	2.14 ± 0.79	1.80 ± 0.51	0.105	0.51
Trunk Flexor 60°·s^−1^ (N·m·kg^−1^)	1.85 ± 0.72	1.68 ± 0.74	0.472	0.23
Trunk Extensor 180°·s^−1^ (N·m·kg^−1^)	1.69 ± 0.61	1.17 ± 0.34	0.002	1.02
Trunk Flexor 180°·s^−1^ (N·m·kg^−1^)	1.42 ± 0.59	1.01 ± 0.42	0.017	0.78

Note: SJ = squat jump; CMJ = counter movement jump; IMTP = isometric mid-thigh pull; PF = peak force; DSI = dynamic strength index; EUR = eccentric utilization ratio; EI = elastic index; D = dominant; ND = nondominant.

**Table 4:** Velocity-specific multiple linear regression models predicting maximal force and stretch-shortening cycle performance.

Outcome	Angular Velocity	Predictors	β	95% CI for β	*p*	VIF
IMTP PF (N·kg^−1^)	60°·s^−1^	Knee strength composite	0.51	0.19 to 0.84	0.003	2.28
		Trunk extensor	0.29	−0.10 to 0.68	0.144	3.36
		Trunk flexor	−0.02	−0.34 to 0.30	0.888	2.20
		Sex	−0.08	−0.33 to 0.16	0.488	1.29
IMTP PF (N·kg^−1^)	180°·s^−1^	Knee strength composite	0.56	0.17 to 0.96	0.006	2.60
		Trunk extensor	−0.03	−0.53 to 0.47	0.897	4.18
		Trunk flexor	0.18	−0.27 to 0.63	0.418	3.39
		Sex	−0.05	−0.34 to 0.24	0.733	1.38
DSI (ratio)	60°·s^−1^	Knee strength composite	−0.13	−0.65 to 0.38	0.604	2.28
		Trunk extensor	0.12	−0.51 to 0.74	0.710	3.36
		Trunk flexor	0.04	−0.46 to 0.55	0.863	2.20
		Sex	0.04	−0.34 to 0.43	0.822	1.29
DSI (ratio)	180°·s^−1^	Knee strength composite	0.01	−0.54 to 0.55	0.977	2.60
		Trunk extensor	0.23	−0.46 to 0.92	0.510	4.18
		Trunk flexor	−0.03	−0.66 to 0.59	0.911	3.39
		Sex	0.14	−0.26 to 0.54	0.478	1.38
EUR (ratio)	60°·s^−1^	Knee strength composite	0.20	−0.27 to 0.67	0.394	2.28
		Trunk extensor	−0.36	−0.93 to 0.21	0.213	3.36
		Trunk flexor	0.52	0.06 to 0.98	0.029	2.20
		Sex	0.03	−0.33 to 0.38	0.881	1.29
EUR (ratio)	180°·s^−1^	Knee strength composite	0.24	−0.30 to 0.78	0.374	2.60
		Trunk extensor	0.14	−0.55 to 0.82	0.689	4.18
		Trunk flexor	−0.23	−0.85 to 0.38	0.446	3.39
		Sex	0.09	−0.31 to 0.48	0.659	1.38
EI (%)	60°·s^−1^	Knee strength composite	0.20	−0.27 to 0.67	0.394	2.28
		Trunk extensor	−0.36	−0.93 to 0.21	0.213	3.36
		Trunk flexor	0.52	0.06 to 0.98	0.029	2.20
		Sex	0.03	−0.33 to 0.38	0.881	1.29
EI (%)	180°·s^−1^	Knee strength composite	0.24	−0.30 to 0.78	0.374	2.60
		Trunk extensor	0.14	−0.55 to 0.82	0.689	4.18
		Trunk flexor	−0.23	−0.85 to 0.38	0.446	3.39
		Sex	0.09	−0.31 to 0.48	0.659	1.38

Note: IMTP = is metric mid-thigh pull; PF = peak force; DSI = dynamic strength index; EUR = eccentric utilization ratio; EI = elastic index; D = dominant; ND = nondominant; β = standardized regression coefficient; CI = confidence interval; VIF = variance inflation factor.

**Table 5:** Goodness-of-fit and diagnostic statistics for the velocity-specific multiple linear regression models predicting maximal force and stretch-shortening cycle performance.

Outcomes	Angular Velocity	R^2^	Adjusted R^2^	SEE (RMSE)	Maximum VIF	Maximum Cook’s Distance
IMTP PF (N·kg^−1^)	60°·s^−1^	0.61	0.56	6.81	3.36	0.12
	180°·s^−1^	0.49	0.44	7.73	4.18	0.15
DSI (ratio)	60°·s^−1^	0.01	−0.09	0.17	3.36	0.10
	180°·s^−1^	0.04	−0.07	0.17	4.18	0.16
EUR (ratio)	60°·s^−1^	0.18	0.08	0.06	3.36	0.29
	180°·s^−1^	0.05	−0.06	0.06	4.18	0.09
EI (%)	60°·s^−1^	0.18	0.08	5.96	3.36	0.29
	180°·s^−1^	0.05	−0.06	6.43	4.18	0.09

Note: IMTP = isometric mid-thigh pull; PF = peak force; DSI = dynamic strength index; EUR = eccentric utilization ratio; EI = elastic index.

## Data Availability

The datasets generated and/or analyzed during the current study are available from the corresponding author upon reasonable request.
